# Developing a Multimodal Screening Algorithm for Mild Cognitive Impairment and Early Dementia in Home Health Care: Protocol for a Cross-Sectional Case-Control Study Using Speech Analysis, Large Language Models, and Electronic Health Records

**DOI:** 10.2196/82731

**Published:** 2026-02-02

**Authors:** Maryam Zolnoori

**Affiliations:** 1 Columbia University Irving Medical Center New York, NY United States

**Keywords:** dementia, fairness, home health care, large language models, mild cognitive impairment, multimodal fusion, natural language processing, screening, speech analysis

## Abstract

**Background:**

Mild cognitive impairment and early dementia (MCI-ED) are frequently unrecognized in routine care, particularly in home health care (HHC), where clinical decisions are made under time constraints and cognitive status may be incompletely documented. Federally mandated HHC assessments, such as the Outcome and Assessment Information Set (OASIS), capture health and functional status but may miss subtle early cognitive changes. Speech, language, and interactional patterns during routine patient-nurse communication, together with information embedded in unstructured clinical notes, may provide complementary signals for earlier identification.

**Objective:**

This protocol describes the development and evaluation of a multimodal screening approach for identifying MCI-ED in HHC by integrating (1) speech and interaction features from routine patient-nurse encounters (verbal communication), (2) large language model–based extraction of MCI-ED–related information from HHC notes and encounter transcripts, and (3) structured variables from OASIS.

**Methods:**

This ongoing cross-sectional case-control study is being conducted in collaboration with VNS Health (formerly Visiting Nurse Service of New York). Eligible participants are adults aged ≥60 years receiving HHC services. Case/control assignment uses a 2-stage process: electronic health record (EHR) prescreening followed by clinician-reviewed cognitive assessment (Montreal Cognitive Assessment and Clinical Dementia Rating) for consented participants without an existing mild cognitive impairment diagnosis. For Aim 1, each participant contributes 3 audio-recorded routine patient-nurse encounters linked to EHR data, including OASIS and free-text clinical notes. Aim 1 extracts acoustic, linguistic, emotional, and interactional features from patient-nurse verbal communication. Aim 2 uses a schema-guided large language model pipeline to extract and normalize MCI-ED–related symptoms, lifestyle risk factors, and communication deficits from HHC notes and encounter transcripts, supported by a human-annotated gold-standard dataset. Aim 3 integrates speech, extracted text variables, and OASIS predictors using supervised machine learning with stratified nested cross-validation; evaluation will include discrimination, calibration, and subgroup performance checks across race, sex, and age.

**Results:**

Between February 2024 and July 2025, a total of 114 HHC patients completed study-administered cognitive assessments and were classified as 55 MCI-ED cases and 59 cognitively normal controls. Audio-recorded patient-nurse encounters had a median duration of 19 (IQR 12-23) minutes and a median of 56 (IQR 31-80) utterances per encounter; nurses contributed more words than patients (median 842, IQR 461-1218 vs median 589, IQR 303-960). In exploratory feasibility analyses, multimodal models integrating speech, interactional features, and structured EHR/OASIS variables outperformed single-source models.

**Conclusions:**

This protocol describes a reproducible multimodal framework for MCI-ED screening in HHC using routinely generated data streams. Initial implementation results support feasibility of data collection and end-to-end processing and suggest potential value of integrating interactional speech features with clinical text and OASIS variables. Final model evaluation, subgroup analyses, and validation will follow the prespecified analytic procedures on the finalized study dataset.

**International Registered Report Identifier (IRRID):**

DERR1-10.2196/82731

## Introduction

Alzheimer disease and Alzheimer disease–related dementias are among the most pressing global public health challenges. In 2021, an estimated 57 million people were living with dementia worldwide, with over 60% residing in low- and middle-income countries and nearly 10 million new cases occurring each year [1]. Dementia is associated with substantial disability, caregiver burden, and rapidly rising health-system costs [2]. In the United States, an estimated 7.2 million adults aged ≥60 years were living with Alzheimer dementia in 2025, underscoring the scale of need in high-income settings as well [3]. Alongside treatment advances and growing interest in risk reduction, global guidance continues to emphasize the importance of timely detection and prevention-oriented interventions across diverse populations and care contexts [4,5].

Despite this urgency, a large proportion of cognitive impairment, particularly mild cognitive impairment (MCI) and early-stage dementia, remains unrecognized or undocumented in routine care, contributing to delayed diagnosis and missed opportunities to tailor care planning [6,7]. In home-based care settings, documentation gaps can be especially consequential because care teams must make clinical decisions in the context of multimorbidity, limited visit time, and incomplete prior cognitive history [8,9]. Recent evidence from skilled home health care (HHC) shows that dementia is frequently undocumented in home health records, illustrating how care transitions and documentation practices can impede recognition of cognitive impairment [10]. These realities motivate scalable screening approaches that can operate within routine workflows, rather than relying solely on specialist evaluation or resource-intensive testing.

Speech and language have emerged as promising noninvasive, low-burden digital biomarkers for cognitive impairment [11-14]. A recent systematic review and meta-analysis focused on MCI specifically concluded that speech-based biomarkers show meaningful diagnostic use, while also highlighting methodological heterogeneity and the need for validation in diverse settings and populations [15]. At the same time, much of the speech-based Alzheimer disease and Alzheimer disease–related dementias detection literature remains anchored in structured elicitation tasks [16] (eg, cookie-theft picture description) and benchmark corpora (eg, DementiaBank-derived shared tasks such as ADReSSo [17]), which enable comparability but may not capture interactional and pragmatic markers expressed during everyday clinical communication [15,18]. Multilingual and cross-cultural work further indicates that generalization across languages and contexts cannot be assumed; for example, multilingual spontaneous speech studies (eg, Italian and Spanish) demonstrate feasibility outside English-centric benchmarks but also reinforce the importance of ecologically valid sampling and external validation [13,19-21].

These limitations are particularly relevant for MCI and early dementia, where impairments can be subtle, context-dependent, and potentially expressed through conversational dynamics (eg, timing, turn-taking balance, and discourse coherence) rather than only through content produced during structured tasks [16]. This motivates studying routine patient-clinician conversations, where interactional features may provide an additional signal for early-stage cognitive change in real-world contexts [22].

A complementary and underused source of early cognitive signals is unstructured clinical documentation, including HHC nursing notes [10,23]. Recent reviews show that natural language processing (NLP) approaches applied to electronic health record (EHR) notes can identify cognitive impairment with strong median performance across studies, but variability in diagnostic criteria, data sources, and external validation remains a key barrier to translation [24]. In parallel, large language model (LLM) methods are increasingly being evaluated for detecting cognitive decline from clinical notes, including large clinical language model approaches (eg, CD-Tron) and comparative studies of LLMs in real-world clinical text [25-29]. These developments suggest that LLM-enabled extraction can help capture both explicit and implicit mentions of symptoms, risk factors, and functional concerns that are inconsistently represented in structured fields, an especially relevant issue in home-based care workflows.

HHC is therefore a compelling setting for scalable, equity-oriented screening because it provides repeated encounters and routinely generates multiple complementary data streams, including standardized assessments (eg, Outcome and Assessment Information Set [OASIS] in US Medicare–certified home health agencies), narrative nursing notes, and patient-nurse verbal communication. Our prior work in HHC has shown that combining structured assessment data with information extracted from clinical notes can improve risk identification (HomeADScreen [12]). More recently, we demonstrated the potential value of leveraging audio-recorded patient-nurse verbal communication as an additional signal beyond EHR data for early cognitive screening in HHC [16]. However, few studies have jointly leveraged (1) standardized home-care assessments, (2) unstructured home-care clinical notes, and (3) routine patient-clinician conversations within a single integrated screening framework for mild cognitive impairment and early dementia (MCI-ED) in home-based care.

Accordingly, this study describes an ongoing protocol to develop and evaluate a multimodal screening approach for identifying MCI and early dementia in HHC using routinely generated data streams: standardized assessment data (OASIS), HHC nursing notes, and audio-recorded patient-nurse verbal communication. We aim to (1) model speech, language, emotion, and interaction patterns from patient-nurse conversations using automated speech analysis, (2) apply NLP/LLM methods to identify MCI/early dementia–related symptoms, lifestyle risk factors, and communication deficits from both clinical notes and verbal communication, and (3) integrate these signals with standardized assessment variables to improve screening performance compared with models based on any single data stream.

## Methods

### Study Setting, Design, and Status

This protocol describes an ongoing cross-sectional case-control protocol in collaboration with VNS Health (formerly Visiting Nurse Service of New York), one of the largest HHC systems in the United States. The study population includes adults aged 60 years and older who receive HHC services from VNS Health. The protocol is designed to develop and evaluate a multimodal screening algorithm for identifying MCI-ED in HHC.

### Participant Recruitment, Eligibility, and Group Allocation

#### Recruitment Focus and Rationale

Aim 1 focuses on modeling speech, language, emotional expression, and interaction patterns during routine patient-nurse encounters (verbal communication) as markers of early cognitive decline in HHC. The primary analytic cohort includes non-Hispanic Black and non-Hispanic White patients receiving HHC services from VNS Health. These groups were selected because they are highly represented in the study setting, enable adequately powered comparisons within a single HHC system, and facilitate evaluation of model performance across racial groups—particularly important given well-documented disparities in dementia diagnosis and care for Black patients.

#### Recruitment Strategy

Potential participants are identified through EHR-based screening and clinician referral workflows within VNS Health. Prespecified EHR indicators are used to identify likely cases (eg, documented symptoms of cognitive decline) and likely controls (no evidence of impairment). Eligible patients are approached during an active episode of HHC. Recruitment is monitored to achieve representation of both racial groups and, when feasible, balance across key characteristics, age, sex as a biological variable, and education.

#### Eligibility, Screening Workflow, and Group Allocation

Eligible participants are aged ≥60 years, plan to receive VNS Health services during the study period, have sufficient English proficiency to communicate independently with HHC nurses, have adequate vision/hearing to complete cognitive testing, and can provide written informed consent. Patients are excluded if they (1) are unable to communicate independently with the HHC nurse in English and (2) have speech or language disorders due to neurological conditions other than MCI-ED (eg, Parkinson disease or seizure disorders). Full eligibility criteria are provided in Multimedia Appendix 1.

We use a 2-stage approach to identify patients for case and control groups. First, we identify potential cases using available *ICD-10* (*International Statistical Classification of Diseases, Tenth Revision*) diagnoses in the EHR (*ICD-10* G31.84 for MCI) and identify potential controls as patients without documented cognitive impairment. Second, all consented participants without an existing MCI diagnosis complete cognitive assessments—the Montreal Cognitive Assessment (MoCA) [30,31] and Clinical Dementia Rating (CDR) [31]—in their homes, administered by a trained research assistant who audio-records responses to support final group assignment.

A study clinician with expertise in cognitive impairment detection reviews the recorded cognitive assessments together with relevant clinical context (medical history and nurse assessment information from OASIS) to confirm group assignment. Based on prespecified criteria, participants are classified as MCI-ED cases when findings are consistent with early cognitive impairment (anticipated CDR 0.5-1 and MoCA ~16-25, with consideration of EHR evidence when available) and as cognitively normal controls when findings are within normal limits (CDR 0 and MoCA ≥26), and there is no EHR evidence of cognitive impairment. Participants meeting criteria for moderate to severe impairment (eg, CDR 2-3 or MoCA <16) are excluded because the protocol focuses on MCI-ED.

After group allocation, patients in both the case and control groups are invited to provide additional consent for the next phase of Aim 1, which includes audio-recording routine patient-nurse encounters.

### Ethical Considerations

This study was reviewed and approved as human participant research by the Columbia University Irving Medical Center Institutional Review Board (Protocol AAAU3168). The study is conducted in collaboration with VNS Health and complies with all applicable institutional, federal, and regulatory requirements for research involving human participants.

Written informed consent is obtained from all participating patients prior to enrollment. Consent includes permission for the administration of cognitive assessments, including the MoCA and the CDR, audio-recording of patient-nurse encounters, and linkage of audio recordings and assessment data with EHR information. HHC nurses also provide informed consent for participation, including consent for audio-recording of patient-nurse encounters. Participants are informed of the study purpose, procedures, potential risks, and their right to withdraw at any time without affecting their care or employment.

All study data are handled in accordance with Health Insurance Portability and Accountability Act and institutional data protection policies. Audio recordings, transcripts, cognitive assessment data, and clinical text are deidentified prior to analysis, with direct identifiers removed. Data are stored on secure, access-controlled servers at Columbia University and VNS Health with role-based permissions and audit logging. Access to identifiable data is restricted to authorized study personnel only. Deidentified datasets are used for analysis, and results are reported in aggregate to minimize the risk of participant reidentification.

Each participating patient receives a US $50 incentive for completion of cognitive assessments (MoCA and CDR) and an additional US $50 incentive for participation in audio-recording of patient-nurse encounters. HHC nurses also receive a US $50 incentive for participation in audio-recording of patient-nurse encounters. Incentives are provided in accordance with institutional review board–approved procedures and are not contingent on study outcomes.

### Data Collection Overview

#### Number of Audio-Recorded Encounters (Patient-Nurse Conversation)

For each enrolled participant, 3 routine patient-nurse encounters are audio-recorded during the HHC episode of care. Recording multiple encounters provides repeated observations to capture within-person variability in speech, language, and interactional patterns across visits, while minimizing participant and clinician burden. When both patient and nurse provide consent, a trained research assistant attends the visit and operates a Saramonic Blink audio-recording device [32], minimizing burden on clinical staff. This portable device, with dual wireless microphones that attach to clothing, provides clear speech transmission to devices like an iPod and offers dual-channel storage.

#### Linking Audio-Recorded Encounters to EHR Data and Clinical Notes

Audio-recorded encounters are linked to EHR data extracted from the VNS Health system, including the OASIS [33,34]—a federally mandated HHC assessment capturing patient health status, functional status, and living arrangements—as well as supplemental structured data (eg, medications) and free-text clinical notes. Free-text notes include visit notes, documenting each nurse encounter, and care coordination notes, capturing communications with other clinicians, physicians, and family members.

### Preliminary Feasibility and Pilot Studies

Prior to this protocol, we conducted a series of pilot studies to establish the feasibility of audio-recording patient-nurse verbal communication and applying automated speech and machine learning methods in the HHC setting [22]. First, we evaluated several commercially available audio-recording devices in laboratory and real-world HHC settings, assessing usability, transcription quality, and acceptability among HHC nurses and patients. Based on System Usability Scale scores and transcription accuracy measured by word error rate, the Saramonic Blink device [35] demonstrated the best overall performance and was selected for use in the current study. Semistructured interviews with HHC nurses and patients further indicated that audio-recording was acceptable and had minimal perceived impact on routine care delivery.

**Figure 1:**
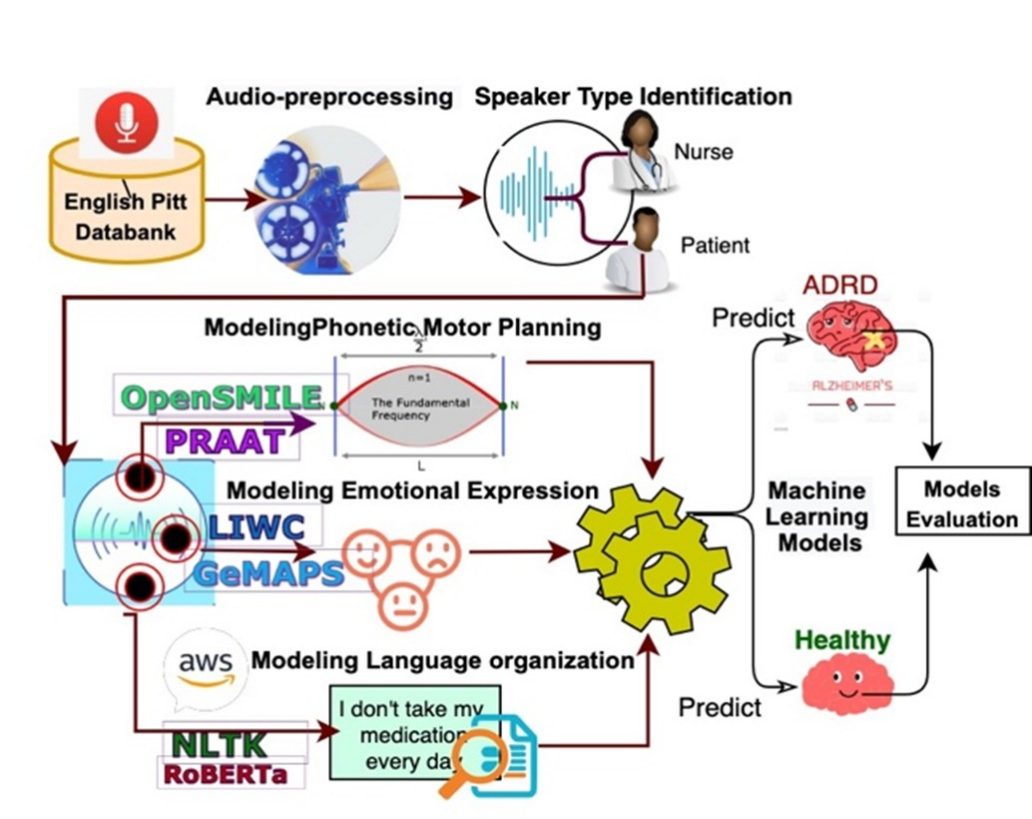
Analytic pipeline for modeling spoken language. ADRD: Alzheimer disease and related dementias; LIWC: Linguistic Inquiry and Word Count; NLTK: Natural Language Toolkit.

In a second pilot study, we demonstrated the feasibility of automated speaker type identification in recorded HHC encounters using machine learning models trained on acoustic and lexical features, achieving satisfactory classification performance [36]. In a third pilot study, we developed and validated an end-to-end analytic pipeline for modeling spoken language in cognitive impairment [11] (Figure 1). The pipeline included (1) audio preprocessing for noise reduction; (2) automated speaker type identification to separate patient and clinician speech; (3) extraction of acoustic features capturing phonetic motor planning and voice characteristics (eg, fluency, frequency/spectral measures, intensity, and instability) using OpenSMILE [37] and PRAAT [38]; (4) modeling of emotional expression using the Geneva Minimalistic Acoustic Parameter Set [39] (GeMAPS) complemented by lexicon-based psycholinguistic markers [40] (using Linguistic Inquiry and Word Count [LIWC]); (5) modeling of language organization using transcript-derived lexical and syntactic measures—using Natural Language Toolkit (NLTK) [41]—and contextual language representations (using distilled RoBERTa [42]); and (6) machine learning–based classification with internal validation. We evaluated this pipeline on a benchmark dataset (DementiaBank [43] “Cookie Theft” picture descriptions) and observed strong discrimination between cognitively impaired and cognitively unimpaired participants, supporting the feasibility of extracting informative speech-derived markers and training predictive models. Collectively, these pilot studies informed the design decisions, data collection procedures, and analytic pipelines in this protocol.

### Analytic Method for Aim 1: Model MCI-ED Patients’ Verbal Communications With Clinicians Using an Automated Speech Analysis System

#### Rationale and Overview

Early cognitive decline affects multiple aspects of spoken communication, including speech motor control, language organization, emotional expression, and social interaction. In HHC settings, these changes are expressed during spontaneous patient-nurse verbal communications rather than structured speech production tasks (eg, reading task). Building on our preliminary feasibility and pilot work, Aim 1 focuses on systematically modeling these communication patterns using an automated speech analysis system. The objective is to extract complementary acoustic, linguistic, emotional, and interactional features from naturally occurring patient-nurse communications that may signal MCI-ED. The analytic framework for Aim 1 consists of 5 components, summarized in Table 1, which together capture core dimensions of speech production and interaction relevant to cognitive decline.

**Table 1:** Modeling mild cognitive impairment and early dementia (MCI-ED) patient-nurse verbal communication in the home health care setting (components 1-4).

Component and domain		Measures
**Component 1: modeling phonetic motor planning**		
	Speech (vocal) fluency	Articulation: number of phonemes per second without hesitation [44]. Speech rate: number of phonemes per second with hesitation [44]. Silent pauses: number of speechless intervals at the beginning of and between words [45]. Within-word disfluency: within-word silent pauses and sound prolongations [46].
	Rhythmic structure of speech	Syllabic intervals: temporal variability in speech [47]. Pairwise variability index: durational variability in successive acoustic-phonetic intervals [48]. Vowel duration: proportion of time of vocalic intervals in a sentence and the standard deviation of inter-vowel intervals [49].
	Frequency and spectral domain	Fundamental frequency: average number of oscillations originating from the vocal folds per second [50]. Formant frequencies (F1-F4): acoustic resonances of the vocal tract due to changes in the positions of vocal organs [51]. Spectral center of gravity: amplitude-weighted mean of harmonic peaks averaged over sound duration [52]. Long-term average spectrum: composite signal representing the spectrum of the glottal source and resonant characteristics of the vocal tract [53]. Mel-frequency cepstral coefficients: energy variations between frequency bands of a speech signal [54].
	Voice instability	Jitter: cycle-to-cycle period variation of successive glottal cycles [55]. Shimmer: cycle-to-cycle amplitude variation of successive glottal cycles [55]. Cepstral peak prominence: measure of periodicity in the speech signal [56].
	Voice quality	Harmonics-to-noise ratio: relative amount of additive noise in the voice signal [50]. Voice breaks: reduced ability in vocal cord execution resulting in voice breaks [57]. Acoustic voice quality index: weighted combination of time-frequency and quefrency-domain metrics developed to measure the severity of dysphonia [58].
	Voice intensity	Hammarberg index: articulatory effort computed as the difference between maximum energy in the 0-2 kHz band and the energy in the 2-5 kHz band [59]. Energy concentration: average spectral frequency [45].
**Component 2: modeling the patient’s emotional expression**		
	Frequency parameters	Pitch: number of vibrations per second produced by the vocal cords [60]. Jitter [55]. Center frequency of formants 1-3. Bandwidth of formants 1-3. Formant frequencies are acoustic resonances of the vocal tract caused by changes in vocal organ positions [51].
	Energy/amplitude	Shimmer. Loudness: estimate of perceived signal intensity from an auditory spectrum [61]. Harmonics-to-noise ratio: relative amount of additive noise in the voice signal [50].
	Spectral parameters	Alpha ratio: ratio of summed energy from 50-1000 Hz and 1-5 kHz. Hammarberg index: measure of articulatory effort [59]. Spectral slope (0-500 Hz and 500-1500 Hz). Formant 1-3 relative energy. Harmonic difference H1-H2: difference between first and second harmonic amplitudes [62]. Harmonic difference H1-A3: difference between H1 and A3 (energy of the highest harmonic in the third formant range) [62]. Spectral flux: difference between the spectra of 2 consecutive frames. Mel-frequency cepstral coefficients: see frequency and spectral domain in component 1.
**Component 3: modeling syntactic, semantic, and pragmatic levels of language organization**		
	Lexical richness	Moving average type-token ratio: total number of unique words divided by the total number of words for each successive fixed-length window [63]. Brunet index: variation in word types marked by part-of-speech tagging relative to the total number of words in a sentence [64]. Honore index: proportion of words used only once relative to the total number of words [65].
	Syntactic level of language organization	Sentence complexity: score computed using a syntactic parse tree [66]. Grammatical errors: identified using a parse tree analyzer [67]. Incomplete (fragment) sentences: identified using an automatic detection algorithm based on syntactic parse trees and part-of-speech tagging [68].
	Semantic fluency	Identification of filled pauses (eg, “um”) in the patient’s spoken language [69-72].
	Patient recall ability	Uncertainty in patient language: computed using the linguistic approximator introduced by Ferson et al [73]. Memory-related terms: proportion of sentences containing memory-related terms relative to the total number of sentences, computed using the NimbleMiner toolkit [74,75]. Question ratio: proportion of interrogative sentences relative to the total number of sentences, identified using the NLTK^a^ Python package [74].
**Component 4: modeling patient-nurse interaction**		
	Patient turns	Continuous block of uninterrupted speech by a single patient. Total number of patient turns indicates frequency of information exchange [76].
	Interactivity	Dialog interactivity: defined as the total number of patient turns divided by the total length of the encounter [76].
	Turn density	Computed using the same parameters specified for lexical richness (component 3).
	Turn duration	Length of time of the patient’s turn; longer durations have been associated with difficulty in turn monitoring in MCI-ED [76].
	Relative timing of turns	Discernible pause rate: proportion of discernible speechless intervals at the start of patient turns relative to total utterances [77]. Cross-over speaking rate: proportion of patient-nurse utterances with cross-over speaking relative to total utterances during the interaction [77].

^a^NLTK: Natural Language Toolkit.

#### Feature Specification and Reproducibility

For reproducibility, all speech- and interaction-based parameters extracted in Aim 1 are explicitly specified in Table 1, organized by analytic component (phonetic motor planning, emotional expression, syntactic and semantic language organization, and patient-nurse interaction). Table 1 provides the operational definition and measurement domain for each parameter. Acoustic features are computed using established toolkits (OpenSMILE [78] and PRAAT [38]); linguistic features are derived from automatically transcribed speech using NLTK, LIWC [79], and distilled RoBERTa; and interactional features are computed from speaker-labeled timestamps generated by Amazon Web Services (AWS) Transcribe.

#### Component 1: Modeling Phonetic Motor Planning

Impairment in phonetic motor planning is a well-documented consequence of neurodegenerative disorders, including MCI-ED, and manifests as reduced articulation precision, altered speech rhythm [44,80,81], and increased disfluency. To characterize these changes, we analyze acoustic parameters across six domains (Table 1, component 1): (1) speech fluency [45,46,82], (2) rhythmic structure [48,49,83], (3) frequency and spectral characteristics [45,84,85], (4) voice instability [45,69,86], (5) voice quality [87-89], and (6) voice intensity [45,90]. These measures quantify temporal and spectral aspects of speech that reflect the patient’s ability to plan and execute vocal motor actions.

#### Component 2: Modeling Emotional Expression

Alterations in emotional expression often develop alongside cognitive decline and can negatively affect communication quality and interpersonal interaction [91,92]. Emotion is conveyed both through nonverbal vocalization and semantic content [93-96]. To model vocal expression of emotion, we use the GeMAPS [39], which captures affect-related changes in autonomic arousal and vocal musculature via frequency-, energy-, and spectral-domain parameters (Table 1, component 2). To capture the semantic expression of emotion, we extract linguistically encoded emotional indicators using the LIWC dictionary. Emotion-related linguistic markers (eg, sadness and anxiety) have been associated with cognitive dysfunction [97] and adverse health outcomes [98].

#### Component 3: Modeling Syntactic, Semantic, and Pragmatic Language Organization

Language impairment in MCI-ED is characterized by reduced lexical diversity, simplified syntax [44,97,99], word-finding difficulties [44,100], and impaired memory-related discourse [44,101], which together contribute to reduced coherence. We model language organization using features in four domains (Table 1, component 3): (1) lexical richness [64,65,102], (2) syntactic complexity and grammaticality [66,103], (3) semantic fluency [104], and (4) patient recall ability [73,74]. Linguistic features are derived from automatically transcribed speech and processed using standard NLP tools. In addition, we will use distilled RoBERTa [42] to generate contextual language representations that capture semantic relationships beyond surface-level lexical features.

#### Component 4: Modeling Patient-Nurse Interaction

Cognitive impairment also affects social communication, including turn-taking, timing, and responsiveness [105,106]. Patients with MCI-ED may show recurrent interactional patterns, such as longer turns, delayed responses, or reduced interactivity [107]. To capture these phenomena, we will model patient-nurse interaction using easily measurable dialogue features [76,77,108] (Table 1, component 4), including patient turn counts, dialog interactivity, turn density, turn duration, and relative timing of turns. These interactional measures reflect how patients engage with clinicians in real-world care encounters and provide information beyond speech content alone.

#### Component 5: System Implementation and Feature Extraction

Acoustic parameters for components 1 and 2 will be computed at the utterance level (continuous blocks of uninterrupted patient speech) using OpenSMILE [37] and PRAAT toolkits [38]. Verbal communications are automatically transcribed using AWS Transcribe, after which linguistic features for component 3 are computed at the encounter level using the NLTK toolkit and distilled RoBERTa. Interactional features for component 4 are derived from AWS Transcribe metadata, including speaker labels and time stamps. All extracted features will be aggregated at the patient level for downstream integration with clinical data in Aim 3.

### Analytic Method for Aim 2: Extraction of MCI-ED–Related Information From Clinical Text Using LLMs

#### Rationale and Overview

Many clinical indicators of MCI-ED—including symptoms, lifestyle risk factors, and communication difficulties—are documented in free-text clinical notes or expressed during patient-nurse conversations [109], but are not captured in structured EHR fields [6,110]. Aim 2 uses LLMs to systematically extract this information from HHC clinical notes and transcripts of patient-nurse verbal communication. The goal is to convert unstructured text into standardized, patient-level variables that can be integrated with speech features (Aim 1) and structured assessment data (OASIS) for multimodal screening (Aim 3).

#### Information Specification and Reproducibility

For reproducibility, all MCI-ED–related information identified in Aim 2 is defined using an information schema summarized in component 1 (Information targets and schema). The schema specifies the target information families (clinical symptoms, lifestyle risk factors, and communication deficits) and associated attributes, and is applied consistently across human annotation and LLM-based identification. MCI-ED–related information is normalized to standard clinical terminologies—Unified Medical Language System (UMLS) [111] concepts, when available—and represented in a structured patient-level format, supporting reproducible integration with Aim 1 features and OASIS variables.

#### Component 1: Information Targets and Schema

We define a schema for the 3 MCI-ED–related risk factor categories, including clinical symptoms, lifestyle risk factors, and communication deficits. For each identified item, the system records (1) the related terms; (2) a normalized clinical concept identifier when available (UMLS [111] concepts); (3) clinically relevant attributes: assertion (present/absent/possible), temporality (current/historical), and experiencer (patient/caregiver); (4) severity and frequency; and (5) duration. The schema is used consistently across clinical notes and transcripts of patient-nurse communication.

#### Component 2: Human-Annotated Gold-Standard Dataset

Using the information schema defined in component 1, we create a human-annotated gold-standard dataset to support LLM adaptation and evaluation. The schema specifies the target information categories (clinical symptoms, lifestyle risk factors, and communication deficits) and associated attributes, including assertion, temporality, and experiencer.

Two trained nurse annotators independently annotate a stratified sample of HHC clinical notes and encounter transcripts according to this predefined schema. The annotation sample is stratified by race, sex, and visit type to ensure representation of diverse documentation patterns. Interannotator agreement is assessed using Cohen κ [112] for each information category and attribute, calculated on double-annotated samples prior to adjudication. Discrepancies are resolved through adjudication meetings to produce a finalized gold-standard dataset. The annotated corpus is subsequently partitioned into training, development, and test sets to enable LLM fine-tuning and unbiased performance evaluation.

#### Component 3: LLM-Based Identification Strategy

We use a hybrid LLM-based strategy that combines prompted extraction and instruction tuning [113], both aligned with the information schema (component 1) and supervised by the human-annotated gold-standard dataset (component 2). First, prompted extraction (baseline): as a baseline approach, we apply structured prompts that explicitly define the 3 information families (clinical symptoms, lifestyle risk factors, and communication deficits) and required attributes (assertion, temporality, and experiencer). Prompts instruct the model to produce schema-compliant JSON outputs and to provide a supporting text span for each identified item to ensure evidence-grounded extraction. This prompted approach provides an interpretable, rapidly adjustable method for early experiments and error analysis. Second, instruction tuning (schema-guided supervised adaptation): to improve reliability on HHC-specific language and documentation patterns, we perform instruction tuning using the training split of the human-annotated gold-standard dataset. Training examples pair the input text (note or transcript segment) with the target output formatted as schema-compliant JSON, including the identified item type, attributes (assertion, temporality, and experiencer), and supporting span. This teaches the model to follow the extraction instructions consistently and to produce outputs that match the schema across diverse note styles and conversational phrasing. Instruction tuning is implemented using parameter-efficient methods (eg, Low-Rank Adaptation [114]/ Quantized Low-Rank Adaptation [114,115]) to reduce computational burden in the secure environment.

#### Component 4: Normalization and Patient-Level Aggregation

For each identified item in the text, we normalize the item (mention) to a standardized clinical concept identifier (UMLS, when available) using a controlled vocabulary lookup supplemented by string similarity matching for common variants. When multiple items map to the same concept within a document, we merge them into a single record while preserving the schema attributes (assertion, temporality, experiencer, and—when present—severity and frequency/duration) and retaining the supporting text spans for traceability. We then aggregate document-level outputs to the patient level to produce predictors for Aim 3. Patient-level variables summarize the presence of each normalized concept and its attributes across the patient’s available HHC notes and encounter transcripts. The final deliverable is a structured patient-level table of normalized MCI-ED–related symptoms, lifestyle risk factors, and communication deficits for integration with OASIS data and Aim 1 speech and interaction features.

#### Component 5: Evaluation, Subgroup Checks, and Quality Control

We evaluate performance against the human-annotated gold-standard dataset using (1) span-level precision/recall/*F*_1_ under exact and overlap matching, (2) attribute performance (assertion, temporality, experiencer, and severity/frequency when applicable), and (3) concept normalization accuracy. We report results overall and stratified by race, sex, and age group to monitor for systematic performance differences. We conduct routine error analysis (eg, common false positives from templated note language, negation errors, or transcript artifacts) and use findings to refine prompts, update normalization resources, and adjust fine-tuning settings. Low-confidence outputs are flagged for targeted review during development to guide iteration and reduce systematic errors.

#### Component 6: Aim 2 Outputs Used in Aim 3

The final Aim 2 deliverable is a structured, patient-level dataset summarizing clinical symptoms, lifestyle risk factors, and communication deficits identified from HHC notes and transcripts, including normalized concept identifiers and clinically meaningful attributes. These variables are used as candidate predictors and complementary signals in the multimodal screening algorithm developed in Aim 3.

### Analytic Method for Aim 3: Development of a Multimodal Screening Algorithm for Identifying MCI-ED in HHC

#### Objective and Overview

The objective of Aim 3 is to develop and evaluate a multimodal screening algorithm for identifying HHC patients with MCI-ED. The algorithm integrates complementary information from three routinely generated data sources: (1) speech and interaction features extracted from patient-nurse communication (Aim 1), (2) MCI-ED–related information identified from clinical notes and transcripts (Aim 2), and (3) structured assessment data from the OASIS.

#### Data Sources and Candidate Predictors

Input variables include (1) acoustic, linguistic, emotional, and interactional features derived from patient-nurse verbal communication (Aim 1); (2) normalized clinical symptoms, lifestyle risk factors, and communication deficits identified from clinical notes and encounter transcripts (Aim 2); and (3) structured OASIS variables capturing sociodemographic characteristics, diagnoses, medications, functional status, and related clinical information.

#### Component 1: Data Preprocessing and Feature Preparation

Prior to model development, we assess data quality and address missingness, inconsistency, and integrity issues using a predefined data quality framework [116]. Continuous variables are transformed and scaled as appropriate to ensure comparability across modalities. To reduce dimensionality and mitigate overfitting in the presence of a large number of candidate predictors, we apply Joint Mutual Information Maximization [117] as a feature selection method. Joint Mutual Information Maximization is selected for its suitability in small to moderate sample settings with high-dimensional data, where it balances relevance to the outcome with redundancy among features.

#### Component 2: Model Development and Multimodal Integration

We develop screening models using supervised discriminative machine learning algorithms that are appropriate for tabular and multimodal clinical data, including logistic regression, support vector machines (SVMs) [118], and ensemble tree-based methods [119-122]. These models are chosen for their interpretability, robustness, and reduced risk of overfitting in clinical datasets. Multimodal integration is performed by combining features from speech, clinical text, and OASIS data within a unified modeling framework. Models are trained to estimate the probability of MCI-ED at the patient level. Temporal aspects of speech-derived features are summarized at the patient level prior to modeling, rather than modeled using complex sequence architectures, to maintain feasibility and stability given sample size considerations.

#### Component 3: Model Training, Validation, and Fairness Assessment

Model training and hyperparameter tuning are conducted using stratified nested cross-validation, with inner loops for parameter selection and outer loops for performance estimation. Model performance is evaluated using the area under the receiver operating characteristic curve (AUC-ROC) and area under the precision-recall curve, along with calibration measures to assess agreement between predicted risk and observed outcomes. To evaluate equitable performance, we assess model metrics stratified by race, sex, and age group. Fairness-related measures, including group-wise differences in sensitivity and specificity and calibration across subgroups, are examined. When systematic performance differences are observed, we explore mitigation strategies such as reweighting or threshold adjustment and reassess model performance.

#### Component 4: Final Model Evaluation and Output

In the final step, the selected model is evaluated on an independent validation dataset to provide an unbiased estimate of performance. The screening algorithm produces a patient-level risk score indicating the likelihood of MCI-ED, which is intended to support clinical awareness and referral for further cognitive evaluation rather than serve as a diagnostic tool. The resulting algorithm and associated feature sets are prepared for downstream evaluation of clinical use and integration into HHC workflows.

## Results

### Initial Study Population and Clinical Characteristics

Between February 2024 and July 2025, we enrolled 114 HHC patients who met eligibility criteria and completed study-administered cognitive assessments. Following standardized review of the cognitive assessments and prespecified group-allocation procedures, 55 participants were classified as MCI-ED cases and 59 as cognitively normal controls. The cohort had a balanced sex distribution (n=58, 51% female) and was racially and ethnically diverse (n=63, 55.3% Black). Most participants were insured by Medicare (n=75, 66%), and 44% (n=50) lived alone.

In descriptive comparisons, participants classified as cognitively impaired had a higher prevalence of urinary incontinence (21/55, 36.8% vs 13/59, 21.4%), anxiety (32/55, 57.9% vs 23/59, 39.3%), and impaired vision (14/55, 26.3% vs 4/59, 7.1%), as well as greater dependence in activities of daily living (41/55, 73.7% completely dependent vs 38/59, 64.3%). These results are reported to characterize the cohort and should be interpreted descriptively rather than as definitive group differences.

Audio-recorded patient-nurse encounters had a median duration of 19 (IQR 12-23) minutes and a median of 56 (IQR 31-80) utterances per encounter. Across encounters, nurses contributed more words than patients (median 842, IQR 461-1218 vs 589, IQR 303-960), consistent with the structure of routine HHC visits and motivating inclusion of interactional features.

### Preliminary Modeling Results

We conducted exploratory modeling analyses to evaluate the feasibility of distinguishing MCI-ED cases from cognitively normal controls using (1) speech-derived measures, (2) clinical text, and (3) structured EHR/OASIS variables, as well as multimodal combinations of these data sources. These analyses are intended to assess feasibility and inform subsequent model refinement and validation, rather than to provide definitive estimates of performance.

#### Speech-Derived Representations

Acoustic and temporal speech features were encoded using SpeechDETECT, including parameters related to phonetic motor planning (Table 1, component 1). Vocal emotion-related cues were encoded using GeMAPS (Table 1, component 2). Linguistic features included handcrafted measures capturing lexical richness, syntactic complexity, and semantic/fluency markers (eg, repetition and filler words; Table 1, component 3), and psycholinguistic indicators were extracted using LIWC 2015. In addition, we evaluated pretrained transformer language models for transcript-based representations.

#### Unimodal Performance

When modeling patient speech alone, DistilBERT achieved the strongest performance among evaluated BERT-based models (*F*_1_=69.39; AUC-ROC=69.36). For clinical notes, BioClinicalBERT yielded the best performance among evaluated language models (*F*_1_=64.29; AUC-ROC=69.17). Among traditional classifiers, a linear SVM performed well using patient speech features (*F*_1_=75.0; AUC-ROC=75.94). Models using structured EHR/OASIS variables achieved their best performance with logistic regression (*F*_1_=75.56; AUC-ROC=79.70).

#### Nurse Speech and Interactional Features

Incorporating nurse speech and interactional measures (Table 1, component 4) resulted in improved discrimination (SVM *F*_1_=85.0; AUC-ROC=86.47), suggesting that patient-nurse interaction captures complementary information beyond patient speech alone.

#### Multimodal Integration

In multimodal analyses integrating speech features, interactional measures, and structured EHR/OASIS variables, the SVM achieved the highest overall performance (*F*_1_=88.89; AUC-ROC=90.23). Examination of model contributions suggested that reduced lexical diversity, longer patient pauses, increased nurse dominance in conversation, selected psycholinguistic markers, and specific EHR variables (eg, non–insulin-dependent diabetes, pressure ulcers, and living alone) contributed to discrimination.

Overall, these results support the feasibility of extracting and integrating multimodal signals for MCI-ED screening in HHC. Final model evaluation, subgroup performance assessment, and fairness analyses will be conducted using the prespecified validation procedures after completion of recruitment and the finalized analytic dataset.

## Discussion

### Overview

This study protocol describes a multimodal screening approach for identifying MCI-ED in HHC using routinely generated data streams. The central hypothesis is that spontaneous patient-nurse conversations, combined with structured HHC assessment data (the federally mandated OASIS instrument) and information extracted from free-text clinical documentation, can provide complementary signals for earlier identification of cognitive impairment than any single data stream alone.

The implementation results reported in this manuscript demonstrate the feasibility of an end-to-end workflow in HHC, including audio capture during routine visits, automated transcription and speaker labeling, extraction of acoustic, linguistic, emotional, and interactional features, and linkage to clinical notes and OASIS variables. Exploratory analyses in the analyzed cohort suggest that incorporating nurse speech and interactional features can improve discrimination beyond patient speech alone, consistent with the premise that conversation structure (eg, timing, pauses, turn-taking balance, and interactivity) contains clinically relevant information. These findings should be interpreted as feasibility and proof-of-concept evidence, rather than definitive estimates of model performance.

A substantial body of prior work has demonstrated that speech markers can differentiate individuals with Alzheimer disease from cognitively unimpaired controls, often using structured or semistructured tasks (eg, picture description, verbal fluency, and reading) collected in controlled environments [13]. While these approaches have been valuable for benchmarking and understanding underlying patterns, they may be less sensitive to the subtle and heterogeneous manifestations of MCI-ED and may not reflect communication behaviors during real-world clinical encounters.

This study extends this literature in 3 important ways. First, it shifts the speech signal from standardized tasks to naturally occurring clinical interactions in HHC, where pragmatic, temporal, and turn-taking patterns can be observed at scale. Second, it models not only patient speech characteristics but also interactional dynamics (including nurse speech), which may reflect clinician adaptation to support patients and/or patient difficulty maintaining conversational flow. Third, it advances a multimodal framework by integrating conversational speech features with (1) structured assessment variables from OASIS and (2) MCI-ED–related information embedded in free-text documentation or spoken conversation but not consistently represented in structured EHR fields. Together, these extensions aim to improve the practical relevance of screening in HHC settings where comprehensive cognitive evaluations may be limited.

Recent advances in minimally invasive approaches to Alzheimer disease detection, including blood-based biomarker testing in symptomatic individuals, reflect increasing clinical emphasis on earlier identification. However, biomarker confirmation alone does not characterize how cognitive decline affects communication during routine care. Changes in speech and language, such as reduced fluency, disrupted discourse organization, and altered vocal control, often emerge early and reflect functional impairment that is not captured by biological measures. The screening approach described here targets this complementary dimension by modeling communication behaviors observed in routine patient-nurse encounters, providing ecologically valid indicators of cognitive change. Integrating speech-based indicators with other clinical information, including biomarker evidence when available, may support a more comprehensive assessment of cognitive decline and its impact on real-world functioning.

### Limitations

Several limitations should be considered when interpreting these results. First, findings are based on data from a single HHC organization and a modest sample, which may limit generalizability and yield performance estimates that are sensitive to sampling variability. Second, audio quality, background noise, and automated transcription/speaker-labeling errors can affect the accuracy of extracted acoustic, linguistic, and interactional features. Third, interactional measures may reflect both patient cognitive-linguistic status and clinician communication style or workflow constraints, which can introduce confounding if not explicitly modeled. Fourth, interactional measures may reflect both patient cognitive-linguistic status and clinician communication style or workflow constraints, which can introduce confounding if not explicitly modeled. Fourth, the protocol focuses on English-speaking participants with sufficient hearing/vision to complete cognitive testing; results may not generalize to other language groups or to patients with sensory limitations, who are common in HHC. Finally, cross-sectional classification does not establish whether speech and interaction markers predict future cognitive trajectories, underscoring the need for longitudinal evaluation.

### Future Directions

Several avenues for future research could strengthen both the scientific rigor and clinical use of this approach. First, prospective longitudinal studies are needed to move beyond cross-sectional classification and evaluate whether speech and interactional markers can predict cognitive decline trajectories or functional deterioration over time. Such studies would clarify whether these features capture progressive change in addition to baseline differences. Second, external validation across diverse HHC agencies, geographic regions, and care delivery models will be essential to assess model transportability and identify when recalibration is necessary. Third, more granular analysis of conversational dynamics could distinguish clinically meaningful interaction patterns—such as repair sequences, prompting behaviors, and topic maintenance difficulties—from structural features that primarily reflect workflow or documentation practices. Fourth, incorporating clinician feedback through human-in-the-loop development cycles can help identify model failure modes, enhance interpretability of predictions, and establish safe deployment thresholds informed by real-world use cases. Finally, pragmatic clinical use trials are needed to determine whether integrating speech-based screening into HHC workflows improves downstream outcomes, including timeliness of formal cognitive evaluation, care plan modifications, and patient safety. Collectively, these efforts would bridge the gap between technical performance and meaningful improvements in care delivery for older adults at risk of cognitive decline.

### Dissemination Plan

Findings will be disseminated through peer-reviewed publications and presentations to clinical and informatics audiences. To support reproducibility while protecting privacy, the study team plans to share (1) detailed feature definitions and extraction procedures, (2) deidentified analytic code and configuration files where permissible, and (3) the annotation schema and evaluation framework for text extraction.

## Appendix

Multimedia Appendix 1 Eligibility criteria.

Multimedia Appendix 2 Peer review report from AGCD-1 - Career Development Facilitating The Transition to Independence Study Section, National Institute on Aging (National Institutes of Health, USA).

## Abbreviations

AUC-ROC: area under the receiver operating characteristic curve
AWS: Amazon Web Services
CDR: Clinical Dementia Rating
EHR: electronic health record
GeMAPS: Geneva Minimalistic Acoustic Parameter Set
HHC: home health care
ICD-10: International Statistical Classification of Diseases, Tenth Revision
LIWC: Linguistic Inquiry and Word Count
LLM: large language model
MCI: mild cognitive impairment
MCI-ED: mild cognitive impairment and early dementia
MoCA: Montreal Cognitive Assessment
NLP: natural language processing
NLTK: Natural Language Toolkit
OASIS: Outcome and Assessment Information Set
SVM: support vector machine
UMLS: Unified Medical Language System
