# Supplementary material for: Developing a Multimodal Screening Algorithm for Mild Cognitive Impairment and Early Dementia in Home Health Care: Protocol for a Cross-Sectional Case-Control Study Using Speech Analysis, Large Language Models, and Electronic Health Records
Source: JMIR Res Protoc. 2026 Feb 2;15:e82731. doi: 10.2196/82731 (PMC12910275; doi:10.2196/82731)
Supplement: Multimedia Appendix 1 [file resprot_v15i1e82731_app1.docx]

## Multimedia Appendix 1 - detailed eligibility criteria

### Inclusion criteria (cases and controls)

1. HHC patients **aged ≥60 years.**
2. Receiving (or scheduled to receive) services from **VNS Health** during the study period.
3. Self-identify as **non-Hispanic Black** or **non-Hispanic White.**
4. Adequate vision and hearing to complete **CDR**and**MoCA.**
5. Sufficient English proficiency to communicate with HHC nurses and complete study procedures in English.
6. Capacity to provide informed consent.

### **Case definition (MCI-ED)**

Participants are classified as cases if MCI-ED is supported by EHR documentation and/or study screening, consistent with early-stage impairment:

- Documented MCI diagnosis in EHR (e.g., **ICD-10 G31.84**) **and/or** study MoCA/CDR consistent with MCI-ED; and
- When no prior diagnosis exists but screening suggests MCI-ED, provider notification and referral for neurologic evaluation occurs; clinician confirmation is used when available.

### Control definition (cognitively normal)

- No documented diagnosis of cognitive impairment in the EHR **and**
- MoCA/CDR scores in the normal range.

### Exclusion criteria (cases and controls)

(Keep your current list and code mappings here; only change the race/ethnicity wording above.)

1. Severe behavioral/psychiatric symptoms (OASIS: **M1710–M1740**; ICD-10 **F20–F29, F40–F48, F60–F69**).
2. Dementia diagnoses other than target early-stage impairment (OASIS: **M1700 score 4–5**; ICD-10 **G30, F00–F09**).
3. Parkinson’s disease (ICD-10 **G20, F02.3, G22, A52.1**).
4. Alcohol/substance abuse within the past 2 years (OASIS **M1036**; ICD-10 **F10–F19**).
5. Severe impairment in speech/oral expression (OASIS **M1230**).
6. Unstable/severely symptomatic cardiovascular disease (OASIS **M1511, M1242**; ICD-10 list as specified).
7. Significant CNS disease (e.g., brain tumor, seizure disorder, subdural hematoma, cranial arteritis).
8. Need for emergent care (OASIS **M2310**).
9. Poorly controlled symptoms affecting daily functioning (OASIS **M1230**).
10. Active systemic cancer within 2 years (ICD-10 list as specified).
11. Need for oxygen supplementation (OASIS **M1410**).
12. Hearing difficulty preventing valid participation (OASIS **M1210**).
13. Progressive vision loss interfering with participation (ICD-10 **H53, H54** and/or OASIS impaired vision).
14. Frequent high-dose opioid use (per clinical review).
15. Moderate-to-severe cognitive impairment (e.g., **CDR 2–3** and **MoCA 0–17**).
16. Unable to communicate in English sufficiently for study procedures.
17. Requires caregiver involvement for communication.
18. Unable to provide informed consent.
