# Supplementary material for: Developing a Multimodal Screening Algorithm for Mild Cognitive Impairment and Early Dementia in Home Health Care: Protocol for a Cross-Sectional Case-Control Study Using Speech Analysis, Large Language Models, and Electronic Health Records
Source: JMIR Res Protoc. 2026 Feb 2;15:e82731. doi: 10.2196/82731 (PMC12910275; doi:10.2196/82731)
Supplement: Multimedia Appendix 2 [file resprot_v15i1e82731_app2.pdf]

SUMMARY STATEMENT
(Privileged Communication)

PROGRAM CONTACT:
Dana Plude
301-435-2309
dana.plude@nih.gov

Release Date: 11/23/2021
Revised Date:

Application Number: 1 K99 AG076808-01

Principal Investigator

ZOLNOORI, MARYAM

Applicant Organization: COLUMBIA UNIVERSITY HEALTH SCIENCES

Review Group: AGCD-1
Career Development Facilitating The Transition to Independence Study Section
NIA-AGCD-1

Meeting Date: 10/14/2021
Council: JAN 2022
Requested Start: 04/01/2022
RFA/PA: PA20-188
PCC: 2BCOGDP

Project Title: Development of a Diagnostic Algorithm for Timely Identification of Patients with Mild Cognitive Impairment and Early-Stage Dementia in the Home Healthcare Setting
SRG Action: Impact Score:46
Next Steps: Visit https://grants.nih.gov/grants/next_steps.htm
Human Subjects: 30-Human subjects involved - Certified, no SRG concerns
Animal Subjects: 10-No live vertebrate animals involved for competing appl.
Gender: 1A-Both genders, scientifically acceptable
Minority: 1A-Minorities and non-minorities, scientifically acceptable
Age: 1A-Children, Adults, Older Adults, scientifically acceptable

---

ADMINISTRATIVE BUDGET NOTE: The budget shown is the requested budget and has not been adjusted to reflect any recommendations made by reviewers. If an award is planned, the costs will be calculated by Institute grants management staff based on the recommendations outlined below in the COMMITTEE BUDGET RECOMMENDATIONS section.

RESUME AND SUMMARY OF DISCUSSION: This application for a Pathway to Independence Award (K99/R00) is submitted by the Columbia University (CU), New York, NY, on behalf of the candidate, Dr. Maryam Zolnoori, requesting five years of support to transition the candidate to an independent career. This application proposes to address barriers to early identification of mild cognitive impairment (MCI) and early-stage dementia (ED) via the development of an innovative algorithm built on a combination of multiple data streams, including electronic health records (EHRs) and audio recorded patient-clinician verbal communication during routine encounters. Dr. Zolnoori is a talented and well-trained candidate with a track record of productivity as evidenced by excellent publications (17 as first author) and several notable awards in recognition of her research work. The candidate has a solid background and training experience in health sciences and clinical informatics including qualitative and quantitative research methodologies. The letters of support provided on behalf of the candidate are enthusiastic. The career development plan (CDP) is well articulated and details the acquisition of skillset in mentoring, training at intramural and extramural workshops and courses, presentations, manuscript preparation and professional development. For each of these activities, formal mentoring is also well organized in the proposed plan. Concerns are noted regarding insufficient details especially on the frequency of meetings with mentors and other team members. Also, some concern with the plans for the K99 phase which is too early before having the outcome of the ongoing feasibility studies. The research plan (RP) is innovative and addresses an important topic of interest focused on the early detection of MCI and ED. The proposed study is novel, and it is built on the Data Information Knowledge Wisdom (DIKW) Framework, in that it will be the first to address the challenges of timely diagnoses of MCI-ED in early patients in Home Health Care (HHC) settings. Using speech analyses and natural language processing algorithms to automatically identify MCI-ED related markers is innovative. However, there are several shortcomings that tempered the enthusiasm. One of the main concerns is the lack of details on the study design, it is unclear whether it focused on cross-sectional or longitudinal research. Also, the consent process could introduce bias and non-referral of suspected MCI cases for proper diagnosis. The recruitment plan is limited and relies on EHRs and it is not clear whether the participants are age and/or gender matched in the proposed study. While consultants are enthusiastic about the work of the Visiting Nurse Service of New York (VNSNY), but insufficient details are provided about these nurses, particularly with regard to their profiles for working with diverse populations with MCI-ED. What specific culturally related training they will receive is not described. Given that speech analysis is a major component of the proposed research, there is a concern with the lack of expertise in speech language pathology. Other concerns include the sample size and lack of details on curtain parameters included in their analysis. The mentoring team, led by Dr. Maxim Topaz with Drs. Julia Hirschberg and James Noble as co-Mentors, is appropriate and will ensure that the candidate has access to the necessary mentorship to complete the proposed work and training. Adding an expertise particularly in speech analysis and biostatistics would improve mentoring plan. Institutional commitment is adequate, offering protected time to the candidate, and the environment is outstanding, offering all necessary resources to complete the proposed work.

TRAINING IN THE RESPONSIBLE CONDUCT OF RESEARCH: Acceptable. The planned activities satisfy the requirement for training in the responsible conduct of research.

DESCRIPTION (provided by applicant): Mild cognitive impairment (MCI) and early-stage dementia (ED) have an adverse impact on elderly patients' quality of life, healthcare utilization, and treatment cost. Despite nationwide efforts for timely diagnosis of MCI and ED, more than 50% of patients remained underdiagnosed and undertreated. This is mostly due to patients' inability to recognize early symptoms, limited availability of biomarkers and clinicians' insufficient time to assess patients for MCI-ED, particularly for patients admitted to the home healthcare (HHC) setting. This K99/R00 will address barriers to early identification of MCI-ED via the development of an innovative algorithm built on a combination of multiple data streams, including data extracted from electronic health records (EHRs)

and audio recorded patient-clinician verbal communication during routine encounters. Our primary goal is to utilize the routinely generated data in the HHC setting, including OASIS (Outcome and Assessment Information Set - a federally required assessment of patients admitted to HHC dataset), HHC nurses' notes, and HHC patient-nurse verbal communication to develop the MCI-ED diagnostic algorithm. The long-term training goal is for Dr. Zolnoori to become an independent investigator conducting a program of research dedicated to mitigating the burden of delayed care for patients with cognitive impairment by developing low- cost, effective informatics solutions. The solutions will take advantage of easily accessible data generated in clinical encounters and will be built on novel data science methods, particularly speech analysis, the focus of her post-doctoral work. Using exceptional resources available from Columbia University and Visiting Nurse Service of New York (VNSNY), the K99 phase of this project will focus on gaining essential competencies and skills in theory and practice of speech analysis and cognitive impairment to quantify properties of MCI-ED patients' verbal communications in interaction with HHC nurses. The R00 phase will focus on the development of a diagnostic algorithm for the early identification of MCI-ED. The specific aims are to 1) model MCI-ED patients verbal communications with HHC nurses using an automated speech analysis system; 2) utilize existing natural language processing algorithms to automatically identify MCI-ED related information, including i) clinical symptoms, ii) lifestyle risk factors, and iii) communication deficits from both HHC clinical notes and patient-nurse verbal communications; 3) develop a sensitive diagnostic algorithm to identify HHC patients with MCI-ED. To accomplish research aims and training goals, an interdisciplinary team of scientists with expertise in speech analysis, cognitive impairment, HHC services, and career development mentorship has been assembled. This project is significant because this algorithm will be built on easily accessible data streams generated during routine patient-nurse encounters. The algorithm has a strong potential to be integrated into HHC clinical workflow to raise clinician's attention to the patient's cognitive functioning for further evaluation and development of proper interventions to reduce the risk of negative outcomes.

PUBLIC HEALTH RELEVANCE: The proposed research is relevant to public health because elderly patients with mild cognitive impairment (MCI) and early-stage dementia (ED) are frequently underdiagnosed, posing significant burden on patients, families and healthcare systems. Development of a novel MCI-ED diagnostic algorithm built on easily accessible data generated in routine patient-clinician encounters has a strong potential to lead to providing timely care for the patients and preventing negative outcomes, particularly emergency department visits, and hospitalization. The proposed study is aligned with the NIA Strategic Directions for Research (2020-2025), "Develop improved approaches for the early detection and diagnosis of disabling illnesses and age-related debilitating conditions."

DISCLAIMER: Please note that the following critiques were prepared by the reviewers prior to the Study Section meeting and are provided in an essentially unedited form. While there is opportunity for the reviewers to update or revise their written evaluation, based upon the group's discussion, there is no guarantee that individual critiques have been updated subsequent to the discussion at the meeting. Therefore, the critiques may not fully reflect the final opinions of the individual reviewers at the close of group discussion or the final majority opinion of the group. Thus, the Resume and Summary of Discussion is the final word on what the reviewers actually considered critical at the meeting.

CRITIQUE 1:

Candidate: 3
Career Development Plan/Career Goals/Plan to Provide Mentoring: 4
Research Plan: 6

Mentor(s), Co-Mentor(s), Consultant(s), Collaborator(s): 4
Environment and Commitment to the Candidate: 1

Overall Impact:
The topic involved in the developmental plan is very important. The development of an innovative algorithm to identify early onset of MCI-ED among home healthcare patients is a step in the right direction. The use of datasets that are routinely generated is a strength of the study. However, the study may be a year or two early. The outcome of ongoing related feasibility studies would have provided meaningful data that will convince reviewers that the proposed plans can be successfully undertaken. The application is also lacking in many vital information that are detailed in other sections of this document and significantly impact the overall quality of the application.

1. Candidate:
Strengths
- The candidate's previous trainings align well with the plan outlined and shows that she is adequately prepared to successfully undertake the proposed study aims.
- The candidate has been involved in many studies that have given her needed experience to successfully navigate the described developmental plan.
- She has authored and co-authored many relevant publications.
- She has demonstrated the potential to work effectively in a multidisciplinary set up.

Weaknesses
- Attention to details in general.

2. Career Development Plan/Career Goals & Objectives:
Strengths
- The career plan is well-conceived and adequate to transition the candidate into an independent researcher whose work will have meaningful contributions better detecting early cognitive decline in older adults.
- The algorithm to be developed from routine communication between patients and clinicians is innovative and has the potential to be a good detector of early onset of cognitive decline.

Weaknesses
- Not waiting for the completion of the ongoing feasibility studies suggests that the K99 phase may be a year too early. The outcome of the feasibility studies is needed to strengthen the application.
- The frequency of meeting with mentors is not well defined.

3. Research Plan:
Strengths
- The use of routinely collected communication between patients and nurses.
- The plan is innovative and can produce important information and knowledge that will potentially lead to early detection of cognitive decline in older adults.

Weaknesses
- Overall, the study approach is confusing.
- It is not clear if this is a longitudinal or cross-sectional study.
- The aspect of participants in this application is very confusing. If the study is on early detection of MCI-ED onset, why include those that have already been diagnosed. If some have been diagnosed, why screen them again?
- It is not clear if participants with and without MCI-ED will be age and gender matched.
- Since patients in VNSNY are older patients who need health care, hence in a healthcare facility, how many will not have one or multiple comorbidities listed as exclusion criteria?

- It is not clear what 17% of patients with symptoms associated with MCI-ED means.
- The study is heavy on data and big data analyses, yet a dedicated biostatistician is not part of the mentoring team.
- It is not clear if what is being studied is motor deficits affecting the communication or cognitive decline despite intact motor capacity.
- The specific parameters that will be included in the analysis were not described.
- Over what time period will the three encounters between the patients and nurses to be included take place?
- If the inclusion and exclusion criteria are specific, helping with identifying, consenting, and audio recording encounters by the candidate may introduce bias.

4. Mentor(s), Co-Mentor(s), Consultant(s), Collaborator(s):
Strengths
- The mentors have the expertise to adequately supervise the candidate during the process.

Weaknesses
- No inclusion of a biostatistical expert in plan that involves mining of huge datasets.
- Insufficient information on frequency of meetings with mentors and collaborators.

5. Environment and Institutional Commitment to the Candidate:
Strengths
- Her institution and Visiting Nurse Service of New York (VNSNY) have the adequate resources needed to support the plan.

Weaknesses
- None noted.

Protections for Human Subjects:
Acceptable Risks and Adequate Protections.
- It is not clear if participants diagnosed with MCI-ED only through administrative cognitive tools can receive targeted interventions without proper medical confirmation of the diagnosis.

Inclusion Plans:
- Sex/Gender: Distribution justified scientifically.
- Race/Ethnicity: Distribution justified scientifically.
- Inclusion/Exclusion Based on Age: Distribution justified scientifically.

Training in the Responsible Conduct of Research:
Acceptable.

Resource Sharing Plans:
Acceptable.

Budget and Period of Support:
Recommend as Requested.

CRITIQUE 2:

Candidate: 1
Career Development Plan/Career Goals/Plan to Provide Mentoring: 4
Research Plan: 5

Mentor(s), Co-Mentor(s), Consultant(s), Collaborator(s): 3
Environment and Commitment to the Candidate: 1

Overall Impact:
The candidate, Dr. Maryann Zolnoori, proposes to develop an MCI-ED diagnostic algorithm in order to identify patients early with cognitive impairment. She proposes that the work will inform a future clinical decision support system for early detection in home health care settings. Her goal is to expand the work for identification of other types of cognitive impairment, such as Alzheimer's disease (AD).

1. Candidate:
Strengths
- Dr. Zolnoori received her PhD in Health Sciences –Clinical Informatics from the University of Wisconsin-Milwaukee, conducted postdoctoral training in 2020 at the Mayo Clinic in Rochester, and is currently in postdoctoral training in Clinical Informatics in Home Health Care.
- She has skillsets in qualitative and quantitative research methodologies and is committed to mitigating the burden of the delayed start of care through multiple data science methods that take advantage of discreet data points from multiple sources, such as speech data, free-text clinical notes, for early identification of patients at risk of health deterioration and negative outcomes.
- She has numerous first author publications and several notable awards for her work, and is very well qualified to carry out the proposed work and to become an independent investigator.

Weaknesses
- None noted.

2. Career Development Plan/Career Goals & Objectives:
Strengths
- The career development plan is well delineated and thorough. It includes mentoring, training at intramural and extramural workshops and courses, presentations, manuscript preparation and application development.
- Feasible short term and long-term goals are presented and are consistent with the FOA, including skill development, writing the R01 and becoming an independent investigator.
- Career development will be structured and guided by an Individual Development Plan.
- There is obvious direct mentoring, guiding, shadowing with mentors.

Weaknesses
- Not provided.

3. Research Plan:
Strengths
- The project benefits from extensive preliminary data, from pre-doctoral and postdoctoral work, from which to build the R00 phase plan.
- The research approach and strategies are sound and will add to an important body of knowledge in ADRD.
- The project, built on the Data Information Knowledge Wisdom Framework, is innovative, and novel, in that it will be the first to address the challenges of timely diagnoses of MCI-ED in early patients in Home Health Care settings.

Weaknesses
- The patient recruitment plan is limited and relies on EHRs from the VNSNY. It was explained how this approach may not provide representation for a broad spectrum of potential patients that would diversify the pool appropriately, if there will be oversampling for certain groups, depending upon the EHR database representation.

- The candidate states that drop-out rates would be reduced by providing incentives. Recruitment studies suggest that this may not be relevant at all and may even be dismissive to some underrepresented groups. A more extensive and culturally relevant recruitment plan is needed for this project.
- The project will require expertise from a biostatistician, which is not included on the team.
- It is unclear how often the team will meet to assure progress.

4. Mentor(s), Co-Mentor(s), Consultant(s), Collaborator(s):
Strengths
- There is an impressive team of mentors and collaborators to assure success.
- All mentors have accomplished scientific and multi-level mentoring careers and are well suited for the success of Dr. Zolnoori.
- Institutional support is excellent, with no teaching and advising commitments expected.

Weaknesses
- A biostatistician would be a great addition to the mentor team.
- While Dr. Zolnoori will meet with her mentor weekly, it is unclear how often the team will meet as an integrated unit to assess her progress.

5. Environment and Institutional Commitment to the Candidate:
Strengths
- The environment at Columbia University Irving Medical Center (CUIMC) is excellent for this project. All mentors and collaborators are highly committed to the success of Dr. Zolnoori.
- The institution is committed to providing support, resources, and protected time for this work.

Weaknesses
- None noted.

Protections for Human Subjects:
Acceptable Risks and Adequate Protections.

Inclusion Plans:
- Sex/Gender: Distribution justified scientifically.
- Race/Ethnicity: Distribution justified scientifically.
- Inclusion/Exclusion Based on Age: Distribution not justified scientifically.

Training in the Responsible Conduct of Research:
Acceptable.
Format:
- The format is appropriate as required.

Subject Matter:
- Subject matter is explicitly detailed.

Faculty Participation:
- Faculty participation is appropriate.

Duration:
- Duration is stated for all training activities.

Frequency:
- Frequency of sessions is stated with mentor, full team and training team.

Resource Sharing Plans:
Acceptable.

Budget and Period of Support:
Recommend as Requested.

CRITIQUE 3:

Candidate: 3
Career Development Plan/Career Goals/Plan to Provide Mentoring: 4
Research Plan: 4
Mentor(s), Co-Mentor(s), Consultant(s), Collaborator(s): 3
Environment and Commitment to the Candidate: 3

Overall Impact:
This a well-written application from a candidate who is well trained in several key areas of the proposed research and has excellent publication record at this stage of her career. The proposed research will develop an algorithm for early identification of MCI-ED using data extracted from electronic health records, audio recorded patient-clinician verbal communication, and Outcome and Assessment Information Set (OASIS) - a federally required assessment of patients admitted to home healthcare (HHC) dataset data. The K99 phase will focus on gaining the missing expertise in the proposed research and modeling verbal communications of MCI-ED patients with clinician using automated speech analysis system (Aim 1). The R00 phase will utilize the existing language processing algorithms to automatically identify MCI-ED related information (clinical symptoms, lifestyle risk factors, and communications deficits) from HHC clinical notes and patient-clinician verbal communications (Aim 2), and develop a diagnostic algorithm to identify HHC patients with MCI-ED based on the three datasets mentioned above. Overall, this is a well written application with clear goals. Developing a reliable predictive algorithm for MCI-ED will make a significant contribution to the healthcare system. Major concerns include a lack of discussion on how different genders would be handled, whether the same or different models be used for both genders, whether the outcome from the models is expected to depend on the gender (mainly due to the verbal component), and the effect of other variables such as the emotional state and mental health on the outcome from the verbal communication.

1. Candidate:
Strengths
- The candidate is well trained in several key areas of the proposed research and has excellent publication record at this stage of her career.

Weaknesses
- While the candidate has strong background in developing decision support systems and natural language processing, no published work on aging and dementia research is a concern. However, gaining expertise in dementia research is proposed as a part of K99 phase and preliminary results involved the use of MCI-ED data, which mitigate this concern.

2. Career Development Plan/Career Goals & Objectives:
Strengths
- A clear path to an independent researcher position is presented. During the pre-doctoral and post-doctoral period, the candidate got a basic training for performing the type of research proposed. The K99 phase will build on this expertise with special focus with expertise in MCI and ED research. The R00 phase will be used to submit an R01 application and transition to an independent position.

- An excellent plan is in place for mentored activities and the role of the mentor and co-mentors in training the candidate in the key areas of the application and guiding her to an independent career.
- Overall, the career goals and plan to achieve these goals are clear.

Weaknesses
- Not provided.

3. Research Plan:
Strengths
- Developing a reliable predictive algorithm for MCI-ED will make a significant contribution to the healthcare system.
- Combining multiple data records to develop predictive algorithm for MCI-ED.
- Overall, the research plan is clearly written.

Weaknesses
- Aim 3 seems descriptive and a little underdeveloped (maybe due to the lack of relevant preliminary data), but it’s a minor concern since this is a career development application.
- A lack of discussion on how different genders would be handled, whether the same or different models be used for both genders, whether the outcome from the models is expected to depend on the gender (mainly due to the verbal component), and the effect of other variables such as the emotional state and mental health on the outcome from the verbal communication is a major concern.
- The above comment also applies to different ethnic groups.
- The success of ML (if any) in identifying diseases or health markers in ADRD or other areas is not sufficiently highlighted.

4. Mentor(s), Co-Mentor(s), Consultant(s), Collaborator(s):
Strengths
- The candidate has a strong team of mentors and co-mentors with significant expertise in all key areas of the proposed research.
- The mentor has strong background in natural language processing and clinical decision support.
- The co-mentors have extensive experience in speech analysis, natural language processing, text analysis, and aging research.

Weaknesses
- Not provided.

5. Environment and Institutional Commitment to the Candidate:
Strengths
- Strong institutional support is in place to train the candidate and ensure the successful completion of the training part of the project.

Weaknesses
- Not provided.

Study Timeline:
Strengths
- Good.

Weaknesses
- None noted.

Protections for Human Subjects:
Acceptable Risks and Adequate Protections.

Inclusion Plans:

- Sex/Gender: Distribution justified scientifically.
- Race/Ethnicity: Distribution justified scientifically.
- Inclusion/Exclusion Based on Age: Distribution justified scientifically.

Training in the Responsible Conduct of Research:
Acceptable.
Format:

- The proposed formats for training in RCR are appropriate.

Subject Matter:

- The institute provides appropriate training on subject matter.

Faculty Participation:

- Faculty participation in RCR is appropriate.

Duration:

- Duration of participation in RCR is appropriate.

Frequency:

- Appropriate frequency for attending events and training on RCR.

Resource Sharing Plans:
Acceptable.

Budget and Period of Support:
Recommend as Requested.

THE FOLLOWING SECTIONS WERE PREPARED BY THE SCIENTIFIC REVIEW OFFICER TO SUMMARIZE THE OUTCOME OF DISCUSSIONS OF THE REVIEW COMMITTEE, OR REVIEWERS' WRITTEN CRITIQUES, ON THE FOLLOWING ISSUES:

PROTECTION OF HUMAN SUBJECTS: ACCEPTABLE. Potential risks and protections of human subjects are adequately described. The committee did not have any concerns.

INCLUSION OF WOMEN PLAN: ACCEPTABLE. Both women and men will be included in this study. The committee did not have any concerns.

INCLUSION OF MINORITIES PLAN: ACCEPTABLE. Both minorities and non-minorities will be included. The review panel did not have any concerns.

INCLUSION ACROSS THE LIFESPAN: ACCEPTABLE. The proposed study focuses on the development of a diagnostic algorithm for identifying elderly patients with MCI and early dementia admitted into home healthcare setting. Children are excluded in this study. The review panel did not have any concerns.

COMMITTEE BUDGET RECOMMENDATIONS: The budget was recommended as requested.

Footnotes for 1 K99 AG076808-01; PI Name: Zolnoori, Maryam

NIH has modified its policy regarding the receipt of resubmissions (amended applications).See Guide Notice NOT-OD-18-197 at https://grants.nih.gov/grants/guide/notice-files/NOT-OD-18-197.html. The impact/priority score is calculated after discussion of an application by averaging the overall scores (1-9) given by all voting reviewers on the committee and multiplying by 10. The criterion scores are submitted prior to the meeting by the individual reviewers assigned to an application, and are not discussed specifically at the review meeting or calculated into the overall impact score. Some applications also receive a percentile ranking. For details on the review process, see http://grants.nih.gov/grants/peer_review_process.htm#scoring.
